# Pervasive Effects of *Wolbachia* on Host Temperature Preference

**DOI:** 10.1128/mBio.01768-20

**Published:** 2020-10-06

**Authors:** Michael T. J. Hague, Chelsey N. Caldwell, Brandon S. Cooper

**Affiliations:** a Division of Biological Sciences, University of Montana, Missoula, Montana, USA; University of Kansas; University of Pittsburgh

**Keywords:** *Drosophila*, host-microbe interaction, symbiosis, thermal adaptation, thermoregulation, *w*Mel

## Abstract

Heritable symbionts can modify a range of ecologically important host traits, including behavior. About half of all insect species are infected with maternally transmitted *Wolbachia*, a bacterial endosymbiont known to alter host reproduction, nutrient acquisition, and virus susceptibility. Here, we broadly test the hypothesis that *Wolbachia* modifies host behavior by assessing the effects of eight different *Wolbachia* strains on the temperature preference of six Drosophila melanogaster subgroup species. Four of the seven host genotypes infected with A-group *Wolbachia* strains (*w*Ri in Drosophila simulans, *w*Ha in *D. simulans*, *w*Sh in Drosophila sechellia, and *w*Tei in Drosophila teissieri) prefer significantly cooler temperatures relative to uninfected genotypes. Contrastingly, when infected with divergent B-group *w*Mau, Drosophila mauritiana prefers a warmer temperature. For most strains, changes to host temperature preference do not alter *Wolbachia* titer. However, males infected with *w*Sh and *w*Tei tend to experience an increase in titer when shifted to a cooler temperature for 24 h, suggesting that *Wolbachia*-induced changes to host behavior may promote bacterial replication. Our results indicate that *Wolbachia* modifications to host temperature preference are likely widespread, which has important implications for insect thermoregulation and physiology. Understanding the fitness consequences of these *Wolbachia* effects is crucial for predicting evolutionary outcomes of host-symbiont interactions, including how *Wolbachia* spreads to become common.

## Abstract (precis)

Microbes infect a diversity of species, influencing the performance and fitness of their hosts. Maternally transmitted *Wolbachia* bacteria infect most insects and other arthropods, making these bacteria some of the most common endosymbionts in nature. Despite their global prevalence, it remains mostly unknown how *Wolbachia* influence host physiology and behavior to proliferate. We demonstrate pervasive effects of *Wolbachia* on *Drosophila* temperature preference. Most hosts infected with A-group *Wolbachia* prefer cooler temperatures, whereas the one host species infected with divergent B-group *Wolbachia* prefers warmer temperatures, relative to uninfected genotypes. Changes to host temperature preference generally do not alter *Wolbachia* abundance in host tissues, but for some A-group strains, adult males have increased *Wolbachia* titer when shifted to a cooler temperature. This suggests that *Wolbachia*-induced changes to host behavior may promote bacterial replication. Our results help elucidate the impact of endosymbionts on their hosts amid the global *Wolbachia* pandemic.

## INTRODUCTION

Heritable symbionts have diverse ecological effects on their hosts. In insects, microbial symbionts influence host reproduction (e.g., cytoplasmic incompatibility) (1, 2), acquisition of nutrients (3–5), tolerance of extreme temperatures (6, 7), and susceptibility to viruses (8, 9). Much less is known about symbionts’ effects on host behavior and their ecological consequences (10–13). On the one hand, symbionts may induce behavioral changes that promote the spread of infection through host populations. Because symbiotic relationships can span a continuum from mutualism to parasitism, behavioral modifications that promote infection spread may not necessarily benefit hosts (2, 14). Parasites, for example, can induce behaviors that are detrimental or lethal to hosts, such as altering host locomotor behavior to increase the probability of parasite transmission (15–20). On the other hand, infected hosts may modify their own behavior in ways that mitigate negative aspects of the infection (16, 21–23), such as a “behavioral chill” thermoregulatory response in which hosts seek cool temperatures to increase their survival probability (24). These behavioral effects represent an important component of how symbionts impact host fitness, which ultimately dictates the evolutionary trajectory of host-symbiont interactions.

Maternally transmitted *Wolbachia* bacteria are the most common endosymbionts in nature, infecting the cells of about half of all insect species, as well as other arthropods (2, 25, 26). *Wolbachia* and host phylogenies are often discordant (27–29), and most *Drosophila* hosts have recently acquired *Wolbachia* via introgressive and/or horizontal transfer (30–32). Maternal transmission occurs in the host germ line, but *Wolbachia* also infects a variety of host somatic cells, including metabolic, digestive, and nervous system tissue (33–35). The fitness consequences of *Wolbachia* in host tissues ultimately determine infection spread, and initial spread from low frequencies requires positive *Wolbachia* effects on host fitness (36–38). Exactly how *Wolbachia* alters components of host fitness is poorly understood (39), even though theoretical and population-level analyses indicate pervasive positive effects on host fitness (1, 31, 37, 40–42).

Symbionts are known to influence host thermal tolerance (7, 43–46), and two recent studies found that Drosophila melanogaster lines infected with the *w*MelCS or *w*Mel *Wolbachia* strain tend to prefer cooler temperatures than uninfected genotypes (47, 48). Modifications to host temperature preference (*T_p_*) have important implications for insects, because ectothermic performance and fitness explicitly depend on temperature (49–55). Because *Wolbachia* infects most insects (2, 25, 26), it is crucial to understand how infections alter host thermoregulation. Few past analyses of insect behavioral thermoregulation have accounted for *Wolbachia* (51, 55, 56).

Differences in *T_p_* between infected and uninfected flies could arise from conflicting physiological requirements of *Wolbachia* and their hosts. *Wolbachia* titer in host bodies is sensitive to temperature fluctuations (57), such that exceedingly cool (<20°C) and warm (>25°C) temperatures can reduce titer and the efficiency of maternal *Wolbachia* transmission (42, 57–63). *Wolbachia*-induced changes to *T_p_* could provide more favorable thermal conditions for bacterial replication in hosts. Alternatively, host-induced changes to *T_p_* could represent a host behavioral response that reduces *Wolbachia* titer to mitigate negative aspects of infection (e.g., behavioral chill). It is still unknown whether observed changes to *T_p_* increase or decrease *Wolbachia* titer (47, 48).

Here, we broadly test for *Wolbachia* effects on host *T_p_* across the D. melanogaster subgroup of flies. Our experiments include seven A-group *Wolbachia*-infected genotypes (*w*Ri in Drosophila simulans, *w*Ha in D. simulans, *w*MelCS in D. melanogaster, *w*Mel in D. melanogaster, *w*Sh in Drosophila sechellia, *w*Yak in Drosophila yakuba, and *w*Tei in Drosophila teissieri) and one B-group *Wolbachia*-infected genotype (*w*Mau in Drosophila mauritiana), which diverged from A-group strains 6 to 46 million years ago (41). We find that hosts infected with four of the A-group *Wolbachia* strains (*w*Ri, *w*Ha, *w*Sh, and *w*Tei) prefer a significantly cooler *T_p_* than uninfected flies of the same host genotype. In contrast, D. mauritiana infected with B-group *w*Mau have a significantly warmer *T_p_*. Unlike previous reports (47, 48), we find no evidence for *w*MelCS or *w*Mel effects on *T_p_* of D. melanogaster, indicating host effects on *T_p_*. Shifting infected adults from an intermediate temperature toward their *T_p_* for 24 h generally does not alter *Wolbachia* titer, but in a few instances, reductions in host *T_p_* seem to promote *Wolbachia* replication. Our results motivate future work on the causes and consequences of *Wolbachia* effects on *T_p_* and other host behaviors.

## RESULTS

### *Wolbachia* infections modify host temperature preference.

**FIG 1:**
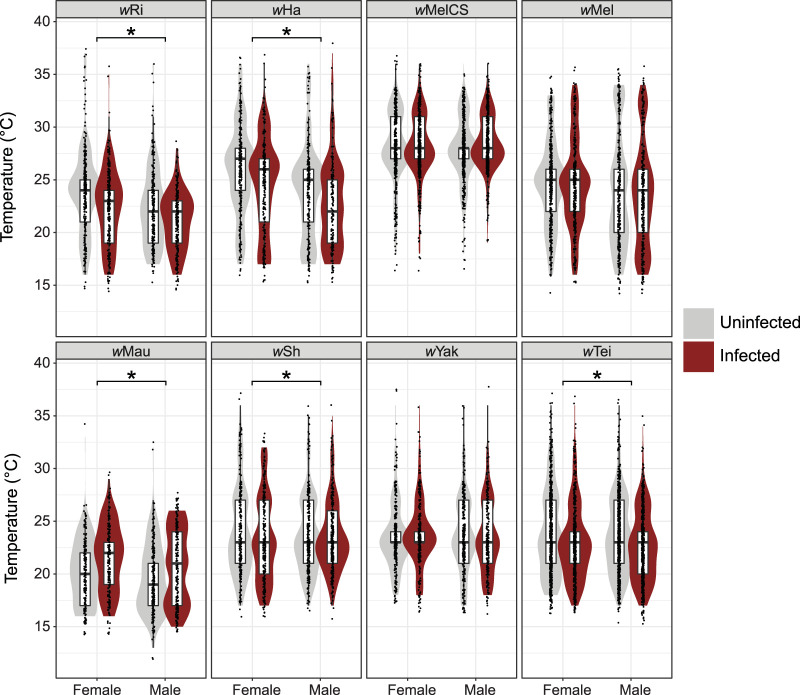
Box plots showing *T_p_* for uninfected and infected flies of each genotype, separated by sex. An asterisk denotes a significant main effect of *Wolbachia* infection on *T_p_* from the GLMMs (Table 1). Individual points are jittered to show overlap. We found a significant main effect of sex on *T_p_* for *w*Ri (χ^2^ = 4.341, *P* = 0.037) and *w*Ha (χ^2^ = 6.907, *P* = 0.009).

**FIG 2:**
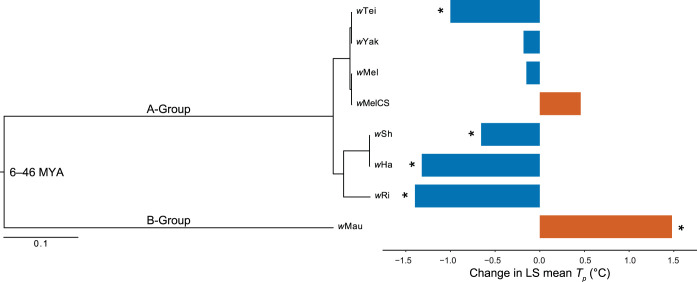
Estimated Bayesian phylogram for A- and B-group *Wolbachia* strains examined in this study. The phylogram was estimated with 214 single-copy genes of identical length in all of the genomes, spanning 181,488 bp. All nodes have Bayesian posterior probabilities of 1. To the right, the change in least-square (LS) mean *T_p_* between uninfected and infected flies is shown for each *Wolbachia* strain. LS means were generated from GLMMs (Table 1), and strains with a significant main effect on *T_p_* are marked with an asterisk. The divergence time estimate (million years ago [MYA]) for A- and B-group *Wolbachia* is from Meany et al. (41).

We used a thermal gradient apparatus to test whether eight different *Wolbachia* strains alter the temperature preference (*T_p_*) of their *Drosophila* host species (see Fig. S1 and Table S1 in the supplemental material). For each strain, we measured the *T_p_* of *Wolbachia*-infected hosts and uninfected flies of the same genotype. In total, we assayed the *T_p_* of 10,401 flies in 347 replicates on the thermal gradient and analyzed our results using generalized linear mixed models (GLMMs) and a Poisson error structure (Table 1 and Fig. 1). *Wolbachia* infection status had a significant main effect on host *T_p_* for five genotypes: *w*Ri-infected D. simulans (χ^2^ = 6.158, *P* = 0.013), *w*Ha-infected *D. simulans* (χ^2^ = 6.148, *P* = 0.013), *w*Mau-infected D. mauritiana (χ^2^ = 7.540, *P* = 0.006), *w*Sh-infected D. sechellia (χ^2^ = 4.531, *P* = 0.033), and *w*Tei-infected D. teissieri (χ^2^ = 8.360, *P* = 0.004) (Table 1). These results were robust to whether the data were analyzed using GLMMs or linear mixed models (LLMs) (Table S2). Of the five *Wolbachia* strains with a significant effect on *T_p_*, all host genotypes infected with A-group *Wolbachia* preferred a cooler temperature than uninfected flies (Fig. 2): *w*Ri-infected *D. simulans* preferred a least-square (LS) mean temperature of 21.72°C ± 1.02°C (±standard error [SE]) compared to 23.12°C ± 1.02°C for uninfected flies, *w*Ha-infected *D. simulans* preferred an LS mean of 23.56°C ± 1.01°C compared to the uninfected mean of 24.89°C ± 1.01°C, *w*Sh-infected *D. sechellia* preferred an LS mean of 23.32°C ± 1.01°C compared to the uninfected mean of 23.98°C ± 1.01°C, and *w*Tei-infected *D. teissieri* preferred an LS mean of 22.7°C ± 1.01°C compared to the uninfected mean of 23.7°C ± 1.01°C. In contrast, *D. mauritana* infected with B-group *w*Mau preferred a warmer LS mean temperature of 21.15°C ± 1.01°C compared to the uninfected mean of 19.67°C ± 1.02°C.

**TABLE 1:** Analysis of host *T_p_* using generalized linear mixed models (GLMMs) and a Poisson error structure^*a*^

Explanatory variable	*w*Ri			*w*Ha			*w*MelCS			*w*Mel		
	Coefficient	χ2	*P* value	Coefficient	χ2	*P* value	Coefficient	χ2	*P* value	Coefficient	χ2	*P* value
Infection status	0.069	6.158	0.013*	0.063	6.148	0.013*	−0.017	1.285	0.257	−0.004	0.031	0.86
Sex	−0.06	4.341	0.037*	−0.07	6.907	0.009*	−0.007	0.224	0.636	−0.046	3.49	0.062
Age	−0.003	0.016	0.898	−0.001	0.02	0.887	−0.019	11.426	0.001*	0.012	2.251	0.134
Run order	0.001	0.013	0.909	0.009	1.002	0.317	0.011	4.914	0.027*	0.005	0.366	0.545
Infection-by-sex	−0.013	0.099	0.754	−0.016	0.186	0.666	0.002	0.005	0.943	0.021	0.368	0.544

Sample size	1,015			857			1,727			1,341		


Explanatory variable	*w*Mau			*w*Sh			*w*Yak			*w*Tei		
	Coefficient	χ2	*P* value	Coefficient	χ2	*P* value	Coefficient	χ2	*P* value	Coefficient	χ2	*P* value
Infection status	−0.091	7.54	0.006*	0.042	4.531	0.033*	0.002	0.007	0.936	0.038	8.36	0.004*
Sex	−0.052	3.175	0.075	0.009	0.238	0.626	−0.01	0.179	0.673	−0.017	1.622	0.203
Age	−0.018	2.686	0.101	0.003	0.13	0.718	0.026	3.633	0.057	−0.012	2.679	0.102
Run order	0.02	3.968	0.046*	0.011	3.187	0.074	0.009	1.32	0.251	0.001	0.083	0.773
Infection-by-sex	0.036	0.68	0.409	−0.029	1.151	0.283	0.011	0.108	0.742	0.011	0.348	0.555

Sample size	818			1,087			10,56			2,500		

a Statistically significant fixed effects at *P* < 0.05 are shown in bold text with asterisks.

10.1128/mBio.01768-20.1 FIG S1 The thermal gradient apparatus is composed of a 44 × 13× 1 cm aluminum plate and a 1-cm-high removable Plexiglas lid. The thermal gradient is subdivided into seven 10 × 6 cm sections (see Table S4 in the supplemental material). Download FIG S1, DOCX file, 0.4 MB. Copyright © 2020 Hague et al. 2020 Hague et al. This content is distributed under the terms of the Creative Commons Attribution 4.0 International license.

10.1128/mBio.01768-20.4 TABLE S1 Genotype IDs for different *Wolbachia*-infected host species used in this study. Download Table S1, DOCX file, 0.01 MB. Copyright © 2020 Hague et al. 2020 Hague et al. This content is distributed under the terms of the Creative Commons Attribution 4.0 International license.

10.1128/mBio.01768-20.5 TABLE S2 Results and sample sizes from the LMM analyses of *T_p_* data. Because the *w*Ha, *w*MelCS, *w*Mel, and *w*Mau data were approximately normally distributed, we analyzed each data set using LMMs. Statistically significant fixed effects at *P* < 0.05 are shown in bold text with asterisks. Download Table S2, DOCX file, 0.01 MB. Copyright © 2020 Hague et al. 2020 Hague et al. This content is distributed under the terms of the Creative Commons Attribution 4.0 International license.

In addition to *Wolbachia* infection status, we found other significant fixed effects on *T_p_*. Sex had a significant main effect on *T_p_* for both the *w*Ri-infected *D. simulans* (χ^2^ = 4.341, *P* = 0.037) and *w*Ha-infected *D. simulans* (χ^2^ = 6.907, *P* = 0.009) (Table 1). For both of these *D. simulans* genotypes, females preferred warmer temperatures than males, regardless of infection status (Fig. 1). For the *w*Ri genotype, infected females preferred an LS mean temperature of 22.37°C ± 1.02°C compared to the uninfected female mean of 23.97°C ± 1.02°C. Infected males preferred an LS mean of 21.07°C ± 1.02°C compared to the uninfected male mean of 22.28°C ± 1.02. For the *w*Ha genotype, infected females preferred an LS mean temperature of 24.41°C ± 1.02°C compared to the uninfected female mean of 25.98°C ± 1.02°C. Infected males preferred an LS mean of 22.75°C ± 1.02°C compared to the uninfected male mean of 23.83°C ± 1.02°C. The GLMMs also revealed a significant effect of fly age on *T_p_* for *w*MelCS-infected D. melanogaster (χ^2^ = 11.426, *P* = 0.001), such that older flies tended to prefer cooler temperatures. Finally, we found that the run order each day had a significant effect on *T_p_* for the *w*MelCS-D. melanogaster (χ^2^ = 4.914, *P* = 0.027) and the *w*Mau-*D. mauritiana* genotypes (χ^2^ = 3.968, *P* = 0.046). In both instances, flies assayed earlier in the day tended to prefer cooler temperatures. This is consistent with prior findings that the *T_p_* of D. melanogaster increases from morning to evening due to a circadian clock (64). In fact, a substrain of the *Canton Special* fly line (our *w*MelCS-D. melanogaster genotype) was specifically shown to have increasing *T_p_* throughout the day (see Materials and Methods for a discussion on *Canton Special* substrains) (64). Circadian clock-dependent temperature preference rhythms help ectotherms maintain homeostasis throughout the day (65). We also detected a main effect of *w*Mau on *D. mauritiana T_p_* only after accounting for run order—*w*Mau had only a marginal effect on *T_p_* when we removed run order from the model (χ^2^ = 3.549, *P* = 0.06).

### *Wolbachia* effects on *T_p_* may exhibit phylogenetic signal.

Notably, hosts infected with A-group *Wolbachia* preferred cooler temperatures, whereas the one species infected with B-group *Wolbachia* preferred a warmer temperature. We conducted a phylogenomic analysis to test whether closely related *Wolbachia* strains exhibit similar effects on host *T_p_*. We generated a *Wolbachia* phylogram and used the change in LS mean *T_p_* of each host genotype to test for phylogenetic signal (Fig. 2). A Pagel’s λ value of 1 is consistent with a model of character evolution that entirely agrees with the phylogeny (i.e., *Wolbachia* effects on host *T_p_* exhibit strong phylogenetic signal), whereas a λ value of 0 indicates that character evolution occurs independently of phylogenetic relationships (66, 67). Our maximum likelihood-fitted λ value was high (λ = 0.778 [0, 0.984]), but not significantly different from a model assuming no phylogenetic signal (likelihood ratio test, *P* = 0.203). Simulations suggest that a much larger number of *Wolbachia* strains are required to statistically distinguish λ ≈ 0.8 from zero (Fig. S2). A simulated *N *= 25 tree had a fitted λ with extremely large confidence intervals (λ = 0.886 [0, 1]), whereas the *N *= 50 tree had a λ estimate that does not overlap with zero (λ = 0.860 [0.376, 0.977]). Unfortunately, far fewer strains exist in laboratory culture, precluding such an analysis. Nevertheless, our finding that most A-group *Wolbachia* decreased host *T_p_* and the one B-group strain increased host *T_p_* hints that divergent *Wolbachia* may have contrasting effects on host behavior.

10.1128/mBio.01768-20.2FIG S2Distribution of maximum likelihood estimates of λ from 1,000 bootstrap replicates. The bootstrap analysis for our *Wolbachia* phylogram (Fig. 2) is shown to the left. To the right are simulated phylogenies with an increasing number of *Wolbachia* strains included (*N *= 25, 50, 100). For simulated trees, character evolution was simulated with our λ estimate of 0.778 using the “sim.bdtree” and “sim.char” functions in the *geiger* R package (147). For each graph, fitted λ values for the original phylogeny are shown above with a vertical dashed line. Note that fitted λ values for the simulated phylogenies differ slightly from λ = 0.778, because “sim.char” uses a Brownian-motion model to simulate character evolution along the phylogeny. Below each graph, the mean estimate of λ from the 1,000 replicates ($\bar{\lambda }$) is shown with associated 95% confidence intervals. The bootstrapping analyses generally show that small phylogenies (*N *= 8, 25) have a large number of near-zero λ values arising by random chance, which increases the uncertainty of parameter estimation. Indeed, small phylogenies are likely to generate near-zero λ values by chance, not necessarily because the phylogeny is unimportant for trait evolution (146). As the number of strains in our analysis increases (*N *= 50, 100), bootstrapped estimates of λ cluster around the true λ value fitted to the original phylogeny. Download FIG S2, DOCX file, 0.1 MB.Copyright © 2020 Hague et al.2020Hague et al.This content is distributed under the terms of the Creative Commons Attribution 4.0 International license.

### 24-h temperature shifts generally do not alter *Wolbachia* titer.

**FIG 3:**
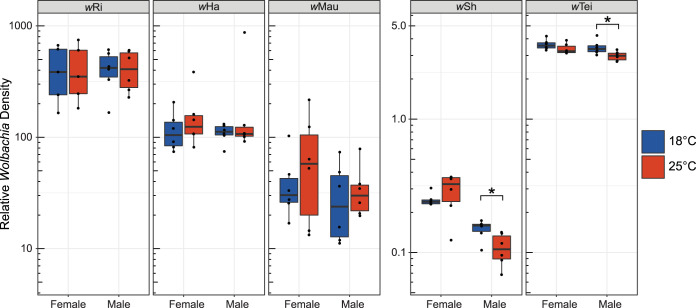
Boxplots of relative *Wolbachia* density from temperature shift experiments for the five *Wolbachia* strains showing main effects on host *T_p_* (Table 1). Relative *Wolbachia* density is shown for virgin females and males shifted to cold (18°C) and warm (25°C) temperatures for 24 h. Graphs are separated into strains with high titer (*w*Ri, *w*Ha, and *w*Mau) and low titer (*w*Sh and *w*Tei). Asterisks denote significant differences in titer between males shifted to 18°C and 25°C based on Wilcoxon rank sum tests at *P* < 0.05.

Truitt et al. speculated that the altered *T_p_* of infected flies represents a host-induced behavior to reduce *Wolbachia* titer and ameliorate the negative effects of infection (47). According to this hypothesis, shifting species infected with A-group *Wolbachia* (*w*Ri, *w*Ha, *w*Sh, and *w*Tei) to a cool temperature should reduce *Wolbachia* titer in host bodies (i.e., behavioral chill), whereas shifting *D. mauritiana* infected with *w*Mau to a warm temperature should reduce *Wolbachia* titer (i.e., behavioral fever). We tested whether shifting infected hosts toward their *T_p_* increases or decreases *Wolbachia* titer (Fig. 3). We reared the five infected genotypes mentioned above at an intermediate temperature of 21.5°C and collected female and male virgins for temperature shift experiments. Adults were maintained as virgins, kept at 21.5°C until they were 3 days old, and then shifted to either a cold (18°C) or warm (25°C) incubator for 24 h, after which we measured *Wolbachia* titer.

For *w*Ri-infected *D. simulans*, *Wolbachia* titer did not differ between the 24-h cold and warm temperature treatments for females (*W * = 12, *P* = 1) or males (*W * = 19, *P* = 0.937). Similarly, for *w*Ha-infected *D. simulans*, titer did not differ between the temperature treatments for females (*W* = 13, *P* = 0.485) or males (*W* = 18, *P* = 1). We also observed no significant difference in titer between temperature treatments for *w*Mau-infected *D. mauritiana* females (*W* = 14, *P* = 0.589) or males (*W* = 14, *P* = 0.589). For *w*Sh-infected *D. sechellia*, we detected no difference in *Wolbachia* titer between females from each temperature treatment (*W* = 13, *P* = 0.485); however, we found that males significantly differed in titer between cold and warm treatments (*W* = 32, *P* = 0.026). Male *D. sechellia* shifted to 18°C had a higher median relative *Wolbachia* density (0.16) than males shifted to 25°C (0.11). This pattern suggests that shifting infected males toward their *T_p_* increases *Wolbachia* titer. We found a similar result for *w*Tei-infected *D. teissieri*. While we detected no difference in *Wolbachia* titer between the treatments for females (*W* = 28, *P* = 0.132), males differed significantly in titer between the cold and warm treatments (*W* = 31, *P* = 0.041). As with *D. sechellia*, male *D. teissieri* shifted to 18°C had a higher median relative *Wolbachia* density (3.36) than males shifted to 25°C (2.98). Importantly, the *w*Sh and *w*Tei results suggest that males shifted to a colder temperature experience an increase in titer; however, these titer increases are not significant at a threshold of *P* = 0.005 after a Bonferroni correction for multiple tests.

## DISCUSSION

Our analyses suggest that *Wolbachia* may generally influence host thermoregulatory behavior. Five of the eight *Wolbachia* strains we assayed had a significant effect on host *T_p_*: *w*Ri in *D. simulans*, *w*Ha in *D. simulans*, *w*Mau in *D. mauritiana*, *w*Sh in *D. sechellia*, and *w*Tei in *D. teissieri*. In contrast to past reports (47, 48), we found no evidence for *w*MelCS or *w*Mel effects on D. melanogaster *T_p_*, which we predict is due to host background effects (see below). Temperature is considered a major ecological factor limiting the distribution of *Drosophila* (55, 56, 68–72) and many other species (73–75). Body temperature is an important determinant of performance and fitness (50, 54, 76–82), and ectotherms depend on thermoregulatory behavior to maintain body temperature within a narrow range (49–53, 55). Given that *Wolbachia* have spread through most insect species and other ectotherms (2, 25, 26), our results motivate additional analyses of *Wolbachia* effects on *T_p_* and thermoregulation of other host taxa.

Interestingly, *Drosophila* species infected with A-group *Wolbachia* generally preferred cooler temperatures, whereas *D. mauritiana* infected with divergent B-group *w*Mau preferred warmer temperatures, suggesting divergent *Wolbachia* effects on host *T_p_*. Our simulations indicate that an unreasonably large number of strains (*N *~ 50) is required to test whether A- and B-group *Wolbachia* effects on *T_p_* exhibit phylogenetic signal (see Fig. S2 in the supplemental material). Indeed, this number of infected species is not currently available to the research community. Nonetheless, our results specifically motivate analyses of whether other B-group *Wolbachia* increase *T_p_*. The only other B-group strains that infect hosts in the D. melanogaster subgroup (*w*No and *w*Sn) almost always occur as coinfections with other *Wolbachia* (41). *w*No co-occurs with *w*Ha in *D. simulans* (83–86), and *w*Sn co-occurs with *w*Sh in *D. sechellia* (85, 87). *D. simulans* and *D*. *sechellia* genotypes singly infected with these B-group *Wolbachia* are currently unavailable. While phylogenetic relationships could be an important determinant of *Wolbachia* effects on host *T_p_*, increases or decreases in *T_p_* could also be idiosyncratic from one host genotype to the next.

Our phylogenetic analysis demonstrates that, in some instances, very closely related *Wolbachia* strains may have different effects on hosts. For example, *w*Tei and *w*Yak diverged only about 1,500 years ago and share very high sequence similarity (0.0039% third-position pairwise differences) (32), yet *w*Tei altered the *T_p_* of *D. teissieri* and *w*Yak had no effect on D. yakuba (Fig. 2). Similarly, *w*Ha and *w*Sh have high sequence similarity according to our analysis (0.00008% third-position pairwise differences) and likely spread recently via introgression (41, 88), yet our mean estimates of titer for *w*Ha in *D. simulans* (157.1) and *w*Sh in *D. sechellia* (0.2) differ by nearly 3 orders of magnitude (Fig. 3). Host background effects may explain why closely related *Wolbachia* can have variable effects on their hosts. Our results from uninfected flies indicate that *T_p_* varies among host genotypes within species. For *D. simulans*, the *T_p_* of the *Wolbachia*-cleared *w*Ri (mean = 23.13°C) and *w*Ha (24.97°C) genotypes was significantly different (Wilcoxon test, *W* = 84398, *P* < 0.001). This was also true for the mean *T_p_* of the uninfected *w*MelCS (27.9°C) and *w*Mel (24.3°C) D. melanogaster genotypes (Wilcoxon test, *W * = 429288, *P* < 0.001). Prior work has similarly found that *T_p_* of D. melanogaster varies in North America along a latitudinal cline (55). Indeed, host genomes seem to modify *Wolbachia* titer (89), maternal *Wolbachia* transmission (90), components of host fitness (91–93), and the strength of cytoplasmic incompatibility (94–96).

We predict that host background effects also underlie our finding that *Wolbachia* does not influence D. melanogaster *T_p_*, in contrast to past reports (47, 48). Arnold et al. (48) found a small, yet statistically significant, reduction in *T_p_* of *w*MelCS-infected D. melanogaster (25.06°C versus 25.78°C for uninfected flies), and Truitt and colleagues (47) found that a *w*MelCS variant identical to our own (according to 720 genes totaling 733,923 bp) reduced D. melanogaster *T_p_* by nearly 4°C. The effect size reported by Truitt et al. (47) is more than two and a half times greater than the largest effect we document here for any strain, and more than five times larger than the reduction in *T_p_* observed by Arnold and colleagues (48). The *w*MelCS variant assayed in Truitt et al. (47) was introduced into the foreign DrosDel *w^1118^* isogenic background using chromosome replacement (97), while Arnold et al. (48) used a standard *Oregon RC* line that was orginally established in the 1920s (8, 98, 99). Our *w*MelCS-infected genotype is a substrain of the *Canton Special* line that was also established in the 1920s (100, 101), and substrains of *Canton Special* can exhibit phenotypic variation due to founder effects and drift (102). It is also worth considering that experimental differences could contribute to differences among *T_p_* studies; for example, differences in the apparatus used to measure *T_p_* (47, 48), fly mating status (103, 104), or statistical approaches could influence *T_p_* estimates. Our analyses accounted for diurnal variation in *T_p_* and host immobilization in the cold (see Materials and Methods), whereas prior analyses did not (47, 48). Regardless, we expect that future analyses of reciprocally introgressed host and *Wolbachia* genotypes will reveal that host and *Wolbachia* genomes, and their interaction, contribute to the variation in *T_p_* observed here.

Our temperature shift experiments indicate that changes to *T_p_* of infected host genotypes generally do not alter *Wolbachia* titer, but in a few instances, reductions in *T_p_* may increase *Wolbachia* replication within host bodies (Fig. 3). *w*Sh-infected *D. sechellia* and *w*Tei-infected *D. teissieri* preferred cooler temperatures than uninfected flies (Fig. 2), and infected males reared at 21.5°C tended to have higher *Wolbachia* titer when shifted to a cold 18°C treatment for 24 h, compared to a warm 25°C treatment (Fig. 3). Moghadam et al. (105) reported a similar effect of cold temperature on *Wolbachia* titer in male D. melanogaster, in which males developed at 13°C had higher microbial diversity and a higher relative abundance of *Wolbachia* than males developed at 23°C and 31°C (based on 16S rRNA sequencing). Our results are consistent with a hypothesis of parasite manipulation, in which *Wolbachia* alters host behavior to seek environmental conditions that promote *Wolbachia* growth (16, 18–20, 22, 23). Importantly, however, we found no temperature-associated increases in titer for *w*Sh- and *w*Tei-infected females or for any other *Wolbachia* strains we assessed. Future work should explore whether changes to male *T_p_* and *Wolbachia* titer alter traits that determine *Wolbachia* infection spread through host populations. Increased *Wolbachia* titer in males is unlikely to affect rates of maternal *Wolbachia* transmission, but perhaps temperature-associated titer increases could alter the strength of cytoplasmic incompatibility caused by males infected with *w*Sh or *w*Tei (85, 87, 95, 106). Other studies have also reported male-biased effects on *Wolbachia* titer (42, 62, 107); for example, our own work demonstrated that maternal transmission of *w*Yak to sons is more efficient than to daughters when *D. yakuba* mothers are reared in cold 20°C conditions (42).

Our findings do not provide support for the hypothesis proposed by Truitt et al. (47) that modifications to *T_p_* represent an adaptive host response (e.g., behavioral chill) to reduce *Wolbachia* titer and mitigate the negative effects of infection (47). In particular, Truitt et al. (47) speculated that *w*MelCS is costly to the host because the strain has a higher titer and growth rate than *w*Mel (97) and that *w*MelCS-infected D. melanogaster prefers colder temperatures to reduce *Wolbachia* titer and limit costly infections. The authors did not measure *w*MelCS titer or estimate host fitness components to test this hypothesis (47), although very recent work has demonstrated that *w*MelCS-infected D. melanogaster has reduced *Wolbachia* titer when raised at 18°C compared to 25°C (108). We found no effects of *w*MelCS or *w*Mel on *T_p_* of D. melanogaster and no evidence that decreases in *T_p_* reduce *Wolbachia* titer for other infected systems (Fig. 3). Nonetheless, the observation that most *Wolbachia*-infected hosts have altered *T_p_* motivates future analyses of host behaviors that might mitigate negative aspects of infection, especially because *Wolbachia* can have costly effects on hosts (37, 109–111). We found no association between changes to *T_p_* and a decrease in adult *Wolbachia* titer, but perhaps infected females seek oviposition sites that reduce the efficiency of *Wolbachia* maternal transmission (51). *Wolbachia* maternal transmission is reduced in relatively cold temperatures in *Drosophila* (42) and hot temperatures in mosquitoes (60, 61). Future work should evaluate whether reductions in host *T_p_* lead to reduced *Wolbachia* titer and maternal transmission downstream over the course of offspring development. For example, mosquito larvae have reduced *w*AlbB titer when reared at temperatures of <20°C (63). Temperature shifts longer than 24 h may also be required to generate reductions in titer, especially if infected hosts seek their *T_p_* throughout their lifecycles.

Our results add to mounting literature showing that temperature is an important abiotic factor mediating interactions between *Wolbachia* and their hosts (112). *Wolbachia* titer seems to be especially sensitive to temperature (42, 58, 60, 61, 63, 113–116). Our 24-h temperature shift experiments suggest that *Wolbachia* titer can change over very short time periods due to environmental conditions. Lau et al. (63) similarly found that *Wolbachia* titer can change within a single host generation, such that cold temperatures (<20°C) reduce *w*AlbB titer in mosquitoes at the larval stage, but then titer rebounds in adulthood when fourth instar larvae are shifted to warmer conditions (>21°C) (63). Temperature-induced changes to *Wolbachia* titer are likely to have cascading effects, given that titer influences other host phenotypes (57). For example, exposure to heat stress is associated with correlated declines in *Wolbachia* titer and the severity of cytoplasmic incompatibility in *w*Mel-transinfected mosquitoes (60, 61). In *Drosophila* hosts, temperature has been shown to modify the strength of cytoplasmic incompatibility (37, 58, 94, 117), maternal transmission (42, 110), and host fitness effects (118–120). Clearly, more work on how temperature influences *Wolbachia*-host interactions is needed.

### Conclusion.

We show that A- and B-group *Wolbachia* bacteria induce changes to host *T_p_* and that short shifts in temperature can increase titer in some *Wolbachia*-infected males. Behavioral changes like these are likely to have fundamental consequences for host physiology and thermoregulation. *Wolbachia* also modifies a range of other ecologically important host traits in *Drosophila* species, including reproduction (1, 2), virus blocking (8, 9, 121, 122), nutrient provisioning (123, 124), and activity levels (12, 17). Given that *T_p_* and many other *Drosophila* traits vary clinally (55, 125), future studies should consider the role of *Wolbachia* in classic *Drosophila* clines (72). For example, *w*Mel infection frequencies (120) and the *T_p_* of D. melanogaster (55) both vary spatially in eastern North America.

Understanding the impact of *Wolbachia* on host performance and fitness is crucial for predicting evolutionary outcomes of *Wolbachia*-host interactions (39). The initial spread of *Wolbachia* through new host populations is driven by beneficial effects on host fitness that cause infections to deterministically spread from low initial frequencies (36–38). Yet, strong positive host effects have not been directly connected to spread in nature for any *Wolbachia*-infected host species (39, 41, 95, 126), although *w*Ri recently evolved to confer a 10% fecundity advantage to *D. simulans* (111). Few data exist for other components of host fitness, but protection from viruses and nutrient provisioning remain candidates for potential host benefits (8, 9, 121–124, 126, 127). Basic research on how *Wolbachia* modifies different components of host fitness, like the effects on *T_p_* reported here, represents a key step to uncovering how *Wolbachia* benefit hosts and spread to become a global pandemic.

## MATERIALS AND METHODS

### Fly lines.

We evaluated eight different *Wolbachia* strains infecting six different species in the D. melanogaster subgroup (see Table S1 in the supplemental material). For two of these host species, we tested multiple *Wolbachia*-infected genotypes: *w*Ri- and *w*Ha-infected *D. simulans* and *w*MelCS- and *w*Mel-infected D. melanogaster. With the exception of the *w*MelCS D. melanogaster line (*Canton S Berkeley*), all our *Wolbachia*-infected genotypes were naturally sampled to form isofemale lines, such that single gravid females were collected from the field and placed individually in vials. *w*MelCS is found only at low frequency in global populations of D. melanogaster (99, 128, 129), because the strain has been largely replaced by a recent sweep of *w*Mel in roughly the last 5,000 years (32, 99, 128, 129). *w*MelCS was originally identified in the common laboratory strain *Canton Special* (99–101), and a substrain (*Canton S Berkeley*) was kindly provided to us by Michael Turelli. All lines were maintained on standard cornmeal medium prior to experiments (Table S3).

10.1128/mBio.01768-20.6 TABLE S3 Fly food recipe for cornmeal media. To the right, the nutritional content of the food is shown based on calculations from https://brodericklab.com/DDCC.php. Download Table S3, DOCX file, 0.01 MB. Copyright © 2020 Hague et al. 2020 Hague et al. This content is distributed under the terms of the Creative Commons Attribution 4.0 International license.

We generated *Wolbachia*-uninfected genotypes by treating each infected line with 0.03% tetracycline for four generations. In the fourth generation, we used PCR to confirm that flies were cleared of *Wolbachia*. We amplified both the *Wolbachia* surface protein (*wsp*) and a second set of primers for the arthropod-specific *28S* rDNA that served as a positive control (41, 95). We also used quantitative PCR (qPCR) on 10 females homogenized together as a more sensitive confirmation of *Wolbachia* removal (see qPCR details below). We then reconstituted the gut microbiome of the tetracycline-cleared flies by rearing them on food where infected males of the same genotype had fed and defecated for the prior 48 h. Tetracycline-cleared flies were given at least three more generations before we conducted experiments to avoid detrimental effects of the antibiotic treatment on mitochondrial function (130).

### Host temperature preference assays.

We assayed the temperature preference (*T_p_*) of each genotype using a thermal gradient apparatus adapted from previous studies (131, 132). The rectangular thermal gradient comprised a 44 × 13 × 1 cm plate of aluminum with a removable Plexiglas lid (see Fig. S1 in the supplemental material). The Plexiglas lid enclosed a 1-cm-high space above the aluminum plate that allows flies to move around on the thermal gradient. We created an air-tight seal between the aluminum plate and the Plexiglas lid using double-sided tape and C-clamps. To keep flies on the temperature-controlled aluminum plate and off the lid, the Plexiglas was coated with Fluon (BioQuip Products), a slick barrier that prevents insects from obtaining a foothold (133, 134). A light-emitting diode (LED) light was placed above the apparatus to ensure that light was evenly distributed across the entire thermal gradient.

All *T_p_* assays were conducted in a cold storage room with a constant temperature of 5°C. A hot plate set at 90°C was placed under one end of the aluminum plate to create a thermal gradient. All experiments began once the apparatus achieved thermal stability after approximately 0.5 h. The aluminum plate was subdivided into seven 10 × 6 cm sections (Fig. S1), and we recorded the temperature at the center of each section using a thermocouple (Digi-Sense Traceable) prior to the start of each experiment. The temperature decreased linearly along the gradient (*R*^2^ = 0.92), ranging from a mean of 34°C at the warmest end (section 1) to 17°C at the coldest end (section 7). Mean temperatures at the center point of each section across all experiments are reported in Table S4.

10.1128/mBio.01768-20.7 TABLE S4 Mean temperature and standard error for each section of the custom-built thermal gradient apparatus, across all 347 experimental replicates in this study. Download Table S4, DOCX file, 0.01 MB. Copyright © 2020 Hague et al. 2020 Hague et al. This content is distributed under the terms of the Creative Commons Attribution 4.0 International license.

The following protocol for our assay was adapted from previous experiments (47, 55, 131, 132). Trial runs revealed that a sample size of 50 to 60 flies allowed flies to distribute across the gradient without overcrowding in preferred temperature ranges, which is consistent with prior studies (47, 131). Flies were reared in a 25°C incubator under a 12-h light:12-h dark light cycle (Pericival model I-36LL) on a standard food diet (Table S3). For each genotype, we collected virgin flies as a batch and separated them into four treatment groups: uninfected females, infected females, uninfected males, and infected males. Flies of each treatment group were separated as virgins in groups of 60 in individual food vials and kept until they were 3 to 5 days old. We selected a single batch each day and ran all four treatment groups separately in a randomized order, such that all flies assayed on a given day were of the same batch and age. All experiments were run between 9 a.m. and 5 p.m. Before each run, we measured the temperature at the center of each section along the gradient and then transferred flies into the apparatus through a small hole located in the middle of the Plexiglas lid where the temperature averaged 22.7°C (Table S4). Flies were allowed to choose their preferred temperatures along the gradient for 30 min (47, 48, 131, 132). At the end of this period, we visually scored the numbers of flies in each section. For our records, we also used a camera mounted above the thermal gradient to take a picture of the distribution of flies in each section. A subset of flies located on the Plexiglas lid were removed from the analysis (132). After each run, the thermal gradient was cleaned with ethanol and allowed to dry. The total number of replicates run for each treatment group ranged from 6 to 21. The final number of flies recorded in each replicate varied due to variation in mortality and the number of flies located on the Plexiglas lid.

For each genotype, we analyzed the *T_p_* data using generalized linear mixed models (GLMMs) and a Poisson error structure in R (135) with the “glmer” function in the *lme4* package (136). We treated the *T_p_* of each fly as the dependent variable and included infection status, sex, an infection-by-sex interaction, fly age (3, 4, or 5 days), and the run order of each replicate over the course of the day (1st, 2nd, 3rd, or 4th) as fixed effects. The replicate identifier (ID) of each run was included as a random effect. We then assessed the significance of fixed effects using an analysis of deviance with chi-squared tests. The *T_p_* data for some genotypes more closely approximated a normal distribution (see Table S2), so we conducted an analogous set of tests using linear mixed models (LMMs) with the “lmer” function in the *lme4* package. Here, we assessed significance of fixed effects using an analysis of variance (ANOVA) with Wald’s chi-squared tests. The LMMs produced qualitatively similar results to the GLMMs, so only results from the GLMMs are presented in the main text.

A preliminary analysis of the data revealed that flies seemed to form a bimodal distribution along the thermal gradient, with one cluster of flies located at the cold end of the gradient (section 7) where temperatures averaged about 17°C (Fig. S3). Given that 17°C generally falls below the average *T_p_* of *Drosophila* species reported in previous experiments (47, 48, 55, 131), we hypothesized that flies were becoming immobilized in section 7 due to the cold temperature (51). A similar phenomenon has been identified for Caenorhabditis elegans in assays of *T_p_*—the movement speed of C. elegans is dependent on temperature, which can leave worms “trapped” in cold sections of a thermal gradient (137). Thus, we removed the putatively immobilized flies in section 7 from each data set and reconducted our analyses. The analyses excluding section 7 are presented in the main text (Table 1); however, including section 7 did not alter our findings of *Wolbachia* effects on *T_p_* (Table S5). We concluded that the data set excluding immobilized flies represents a more biologically accurate measure of *T_p_* for each genotype.

10.1128/mBio.01768-20.3 FIG S3 Box plots showing temperature preference (*T_p_*) for uninfected and infected flies of each genotype when the coldest section of the thermal gradient (section 7) is included. Individual points are jittered to show overlap. Download FIG S3, DOCX file, 0.5 MB. Copyright © 2020 Hague et al. 2020 Hague et al. This content is distributed under the terms of the Creative Commons Attribution 4.0 International license.

10.1128/mBio.01768-20.8 TABLE S5 Results and sample sizes from the GLMM and LMM analyses of *T_p_* data, including the flies located in the coldest section of the thermal gradient apparatus (section 7). Statistically significant fixed effects at *P* < 0.05 are shown in bold text with asterisks. Download Table S5, DOCX file, 0.02 MB. Copyright © 2020 Hague et al. 2020 Hague et al. This content is distributed under the terms of the Creative Commons Attribution 4.0 International license.

### *Wolbachia* sequencing and phylogenomic analysis.

We conducted a phylogenomic analysis to characterize the evolutionary relationships among *Wolbachia* strains included in this study. Hosts infected with A-group *Wolbachia* (*w*Ri, *w*Ha, *w*Sh, and *w*Tei) preferred cooler temperatures, whereas *D. mauritiana* infected with B-group *w*Mau preferred a warmer temperature. Therefore, we used a *Wolbachia* phylogram to test whether these *Wolbachia* effects on host *T_p_* exhibit phylogenetic signal. We obtained *Wolbachia* sequences from publicly available genome assemblies, which included *w*Ri (138), *w*Ha (139), *w*Mau (41), and *w*Yak and *w*Tei (32). We also obtained raw Illumina reads for a *w*Sh-infected *D. sechellia* individual from a previously published data set (NCBI:SRA accession no. SRX3029362) (140). Importantly, two divergent *Wolbachia* strains may infect *D. sechellia*: A-group *w*Sh and B-group *w*Sn. In nature, *w*Sh singly infects some individuals, but it also occurs as a coinfection with *w*Sn (85). We confirmed that our *D. sechellia* genotype (*PmuseumbananaI*) is singly infected with *w*Sh using qPCR primers described below, which can distinguish between A-group and B-group *Wolbachia*. Finally, we sequenced our *w*MelCS- and *w*Mel-infected D. melanogaster genotypes (*Canton S Berkeley* and *PC75*, respectively) to compare the sequence similarity of our variants of these strains to those used in the prior assay of *T_p_* by Truitt et al. (47, 97).

Tissue samples for genomic DNA were extracted using a DNeasy Blood & Tissue kit (Qiagen). DNA quantity was tested on a Nanodrop (Implen), and total DNA was quantified by Qubit fluorometric quantitation (Invitrogen). DNA was cleaned using Agencourt AMPure XP beads (Beckman Coulter, Inc.) following the manufacturer’s instructions, and eluted in 50 μl of 1× TE (Tris-EDTA) buffer for shearing. DNA was sheared using a Covaris E220 Focused Ultrasonicator (Covaris Inc.) to a target size of 400 bp. We prepared libraries using NEBNext Ultra II DNA Library Prep with Sample Purification beads (New England BioLabs). Final fragment sizes and concentrations were confirmed using a TapeStation 2200 system (Agilent). We indexed samples using NEBNext Multiplex Oligos for Illumina (Index Primers Set 3 and Index Primers Set 4), and 10 μl of each sample was shipped to Novogene (Sacramento, CA, USA) for sequencing using Illumina HiSeq 4000, generating paired-end 150 bp reads.

Reads were trimmed using Sickle version 1.33 (141) and assembled using ABySS version 2.0.2 (142). *K* values of 71, 81, and 91 were used, and scaffolds with the best nucleotide BLAST matches to known *Wolbachia* sequences with E values less than 10^−10^ were extracted as the draft *Wolbachia* assemblies. For each genotype, we chose the assembly with the highest *N*_50_ and the fewest scaffolds (Table S6). The *w*MelCS, *w*Mel, and *w*Sh genomes, along with the five previously published genomes were annotated using Prokka version 1.11, which identifies homologs to known bacterial genes (143). To avoid pseudogenes and paralogs, we only used genes present in a single copy with no alignment gaps in all of the genome sequences. Genes were identified as single copy if they uniquely matched a bacterial reference gene identified by Prokka. By requiring all homologs to have identical length in all of the *Wolbachia* genomes, we removed all loci with indels. A total of 214 genes totaling 181,488 bp met these criteria.

10.1128/mBio.01768-20.9 TABLE S6 The scaffold count, *N*_50_, and total assembly size of each *Wolbachia* assembly. Download Table S6, DOCX file, 0.01 MB. Copyright © 2020 Hague et al. 2020 Hague et al. This content is distributed under the terms of the Creative Commons Attribution 4.0 International license.

We also repeated this analysis to include the *w*MelCS and *w*Mel genomes used in Truitt et al. (47). Here, we restricted our analysis to only *w*MelCS and *w*Mel *Wolbachia*, with the goal of comparing sequence similarity between the variants used in this study to those from Truitt et al. (47). Given that many loci accumulate indels over time, the number of loci included in this analysis of *w*Mel-like *Wolbachia* was relatively high, with a total of 720 genes totaling 733,923 bp that met our criteria. Based on these 720 genes, our *w*MelCS variant infecting the *Canton S Berkeley* genotype was identical to the *w*MelCS variant used in Truitt et al. (47). Our *w*Mel variant infecting the *PC75* genotype was also highly similar to *w*Mel used in Truitt et al. (47), with only 0.000016% third-position pairwise differences (only 4 out of 244,641 third-codon positions).

We estimated a Bayesian phylogram of the 214 genes from the eight different *Wolbachia* strains using RevBayes 1.0.8 under the general tree reversible GTR + Γ model partitioned by codon position (144). Four independent runs were performed for each phylogenetic tree we estimated, and in each instance, all four runs converged on the same topology. All nodes were supported with Bayesian posterior probabilities of 1.

We used the resulting phylogram to test whether *Wolbachia* effects on host *T_p_* exhibit phylogenetic signal. For each genotype, we extracted the least-square (LS) mean *T_p_* for infected and uninfected flies from the GLMMs and then used the change in LS mean *T_p_* as a continuous character to calculate the maximum likelihood value of Pagel’s lambda (λ) (67). We used a likelihood ratio test to compare our fitted value of λ to a model assuming no phylogenetic signal (λ = 0) using the “phylosig” function in the R package *phytools* (145). We also employed a Monte Carlo-based method to generate 95% confidence intervals surrounding our λ estimate using 1,000 bootstrap replicates in the R package *pmc* (146). To evaluate whether larger phylogenies increase the accuracy of λ estimation, we simulated trees with an increasing number of *Wolbachia* strains (*N *= 25, 50, and 100) and our λ estimate of 0.778 using the “sim.bdtree” and “sim.char” functions in the *geiger* R package (147). We then reestimated confidence intervals surrounding λ using the larger simulated trees. See Fig. S2 for an extended description of the simulations.

### Host temperature shift experiments.

We tested whether shifting infected hosts toward their *T_p_* increases or decreases *Wolbachia* titer. We reared the five infected host genotypes with altered *T_p_* at an intermediate temperature of 21.5°C. We separated female and male virgins, kept them at 21.5°C until they were 3 days old, and then shifted them to either a cold (18°C) or warm (25°C) incubator for 24 h. Flies were separated by sex and maintained in groups of 40 in individual food vials throughout the course of the experiment. Following 24 h of the cold/warm temperature treatment, flies were frozen in a −80°C freezer for subsequent analysis of *Wolbachia* titer.

We used qPCR to compare *Wolbachia* titer in flies shifted to 18°C versus 25°C. Flies from each temperature treatment were homogenized together in groups of 10. The final samples included six biological replicates for each sex and temperature treatment. DNA was extracted using a DNeasy Blood & Tissue kit (Qiagen). Preliminary analyses indicated that our extractions contained DNA quantities that are well within the recommended range for PowerUp SYBR green Master Mix (Thermo Fisher Scientific) used in our qPCRs. We used a Stratagene Mx3000P (Agilent Technologies) to amplify *Drosophila*- and *Wolbachia*-specific loci. In order to quantify the titers of the five different *Wolbachia* strains, we utilized multiple combinations of *Drosophila* and *Wolbachia* qPCR primers (Table S7). Efficiency curves were generated to confirm that each primer pair had adequate efficiency. All qPCRs were amplified using the following cycling conditions: 50°C for 2 min, 95°C for 2 min, and then 40 cycles, with one cycle consisting of 95°C for 15 s, 58°C for 15 s, and 72°C for 1 min. We used the average cycle threshold (*Ct*) value of three technical replicates for each sample. We estimated relative *Wolbachia* density as $2^{\Delta \mathrm{Ct}}$, where Δ*Ct* = *Ct*_host_ – *Ct_Wolbachia_* (148). We then used a Wilcoxon rank sum test to assess differences in titer between flies shifted to 18°C and 25°C.

10.1128/mBio.01768-20.10 TABLE S7 qPCR primers used to measure relative *Wolbachia* density in temperature shift experiments. Download Table S7, DOCX file, 0.01 MB. Copyright © 2020 Hague et al. 2020 Hague et al. This content is distributed under the terms of the Creative Commons Attribution 4.0 International license.

### Data availability.

Genome assemblies are deposited on GenBank (BioProject accession no. PRJNA658309). All other data are available on Dryad (https://doi.org/10.5061/dryad.j9kd51c8r).
