# Supplementary material for: Pervasive Effects of Wolbachia on Host Temperature Preference
Source: mBio. 2020 Oct 6;11(5):e01768-20. doi: 10.1128/mBio.01768-20 (PMC7542361; doi:10.1128/mBio.01768-20)
Supplement: FIG S1 [file mBio.01768-20-sf001.docx]

**
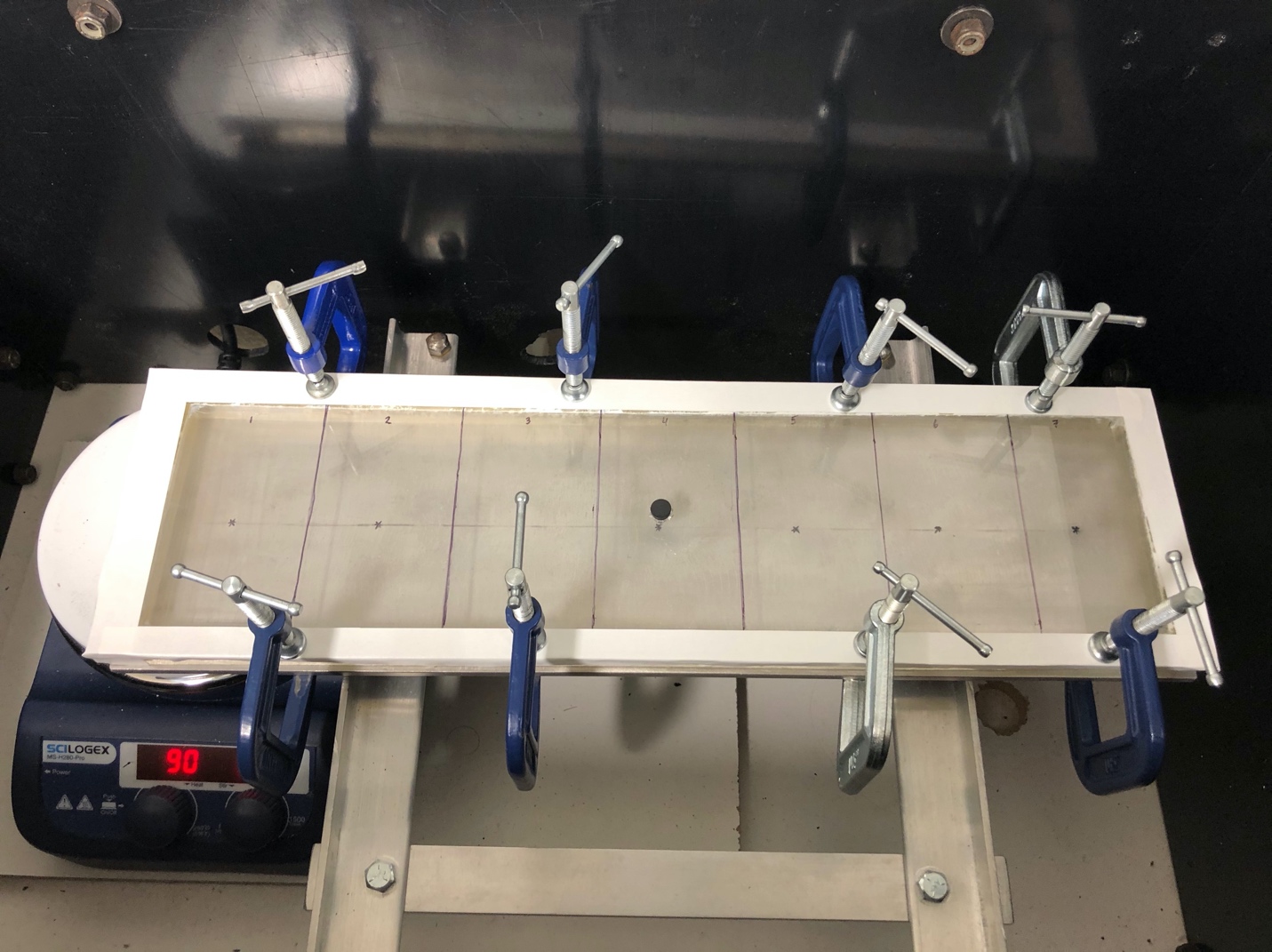
**

**Supplemental Figure S1.** The thermal gradient apparatus is composed of a 44 x 13 x 1 cm aluminum plate and a 1 cm-high removable Plexiglas lid. The thermal gradient is subdivided into seven 10 x 6 cm sections (Supplemental Table S4)
