# Supplementary material for: Pervasive Effects of Wolbachia on Host Temperature Preference
Source: mBio. 2020 Oct 6;11(5):e01768-20. doi: 10.1128/mBio.01768-20 (PMC7542361; doi:10.1128/mBio.01768-20)
Supplement: TABLE S1 [file mBio.01768-20-st001.docx]

**Supplemental Table S1.** Genotype IDs for different *Wolbachia*-infected host species used in this study.

| **Species** | ***Wolbachia*** | **Genotype ID** | **Location** | **Approximate Collection Date** |
| --- | --- | --- | --- | --- |
| *D. simulans* | *w*Ri | *Riv84* | Riverside, California, USA | 1984 |
| *D. simulans* | *w*Ha | *Car5* | Hawaii, USA | 2016 |
| *D. melanogaster* | *w*MelCS | *Canton S Berkeley* | Canton, Ohio, USA | 1920s |
| *D. melanogaster* | *w*Mel | *PC75* | Panama City, Panama | 2012 |
| *D. mauritiana* | *w*Mau | *mauR31* | Mauritius | 2006 |
| *D. sechellia* | *w*Sh | *PmuseumbananaI* | Praslin, Seychelles | 2012 |
| *D. yakuba* | *w*Yak | *B13L5* | Bioko, Equatorial Guinea | 2013 |
| *D. teissieri* | *w*Tei | *B13L11* | Bioko, Equatorial Guinea | 2013 |
