# Supplementary material for: Pervasive Effects of Wolbachia on Host Temperature Preference
Source: mBio. 2020 Oct 6;11(5):e01768-20. doi: 10.1128/mBio.01768-20 (PMC7542361; doi:10.1128/mBio.01768-20)
Supplement: TABLE S2 [file mBio.01768-20-st002.docx]

**Supplemental Table S2.** Results and sample sizes from the LMM analyses of *T_p_* data. Because the *w*Ha, *w*MelCS, *w*Mel, and *w*Mau data were approximately normally distributed, we analyzed each dataset using LMMs. Statistically significant fixed effects at *P* < 0.05 are marked in bold text with asterisks.

|  | ***w*Ri** | | | ***w*Ha** | | | ***w*MelCS** | | | ***w*Mel** | | |
| --- | --- | --- | --- | --- | --- | --- | --- | --- | --- | --- | --- | --- |
| **Explanatory variable** | **coefficient** | **χ2** | ***P* value** | **coefficient** | **χ2** | ***P* value** | **coefficient** | **χ2** | ***P* value** | **coefficient** | **χ2** | ***P* value** |
| Infection Status | 1.607 | 5.082 | **0.024*** | 1.576 | 5.201 | **0.023*** | -0.492 | 1.065 | 0.302 | -0.101 | 0.027 | 0.871 |
| Sex | -1.36 | 3.544 | 0.06 | -1.64 | 5.438 | **0.02*** | -0.197 | 0.155 | 0.694 | -1.125 | 3.178 | 0.075 |
| Age | -0.16 | 0.092 | 0.761 | -0.048 | 0.058 | 0.81 | -0.574 | 9.683 | **0.002*** | 0.316 | 2.165 | 0.141 |
| Run Order | 0.026 | 0.013 | 0.909 | 0.167 | 0.533 | 0.465 | 0.314 | 3.613 | 0.057 | 0.103 | 0.264 | 0.608 |
| Infection * Sex | -0.383 | 0.141 | 0.707 | -0.457 | 0.206 | 0.65 | 0.023 | 0.001 | 0.974 | 0.528 | 0.357 | 0.55 |
| Sample Size | 1015 |  |  | 857 |  |  | 1727 |  |  | 1341 |  |  |
|  |  |  |  |  |  |  |  |  |  |  |  |  |
|  | ***w*Mau** | | | ***w*Sh** | | | ***w*Yak** | | | ***w*Tei** | | |
| **Explanatory variable** | **coefficient** | **χ2** | ***P* value** | **coefficient** | **χ2** | ***P* value** | **coefficient** | **χ2** | ***P* value** | **coefficient** | **χ2** | ***P* value** |
| Infection Status | -1.871 | 5.086 | **0.024*** | 1.019 | 4.336 | **0.037*** | 0.035 | 0.003 | 0.958 | 0.821 | 6.718 | **<0.001*** |
| Sex | -1.088 | 2.127 | 0.145 | 0.261 | 0.314 | 0.575 | -0.248 | 0.143 | 0.705 | -0.398 | 1.555 | 0.212 |
| Age | -0.349 | 1.509 | 0.219 | 0.072 | 0.109 | 0.742 | 0.608 | 2.814 | 0.093 | -0.271 | 2.535 | 0.111 |
| Run Order | 0.456 | 3.065 | 0.08 | 0.253 | 2.673 | 0.102 | 0.223 | 1.073 | 0.3 | 0.02 | 0.041 | 0.84 |
| Infection * Sex | 0.793 | 0.507 | 0.477 | -0.799 | 1.386 | 0.239 | 0.291 | 0.097 | 0.755 | 0.328 | 0.552 | 0.458 |
| Sample Size | 818 |  |  | 1087 |  |  | 1056 |  |  | 2500 |  |  |
