# Supplementary material for: Pervasive Effects of Wolbachia on Host Temperature Preference
Source: mBio. 2020 Oct 6;11(5):e01768-20. doi: 10.1128/mBio.01768-20 (PMC7542361; doi:10.1128/mBio.01768-20)
Supplement: FIG S2 [file mBio.01768-20-sf002.docx]

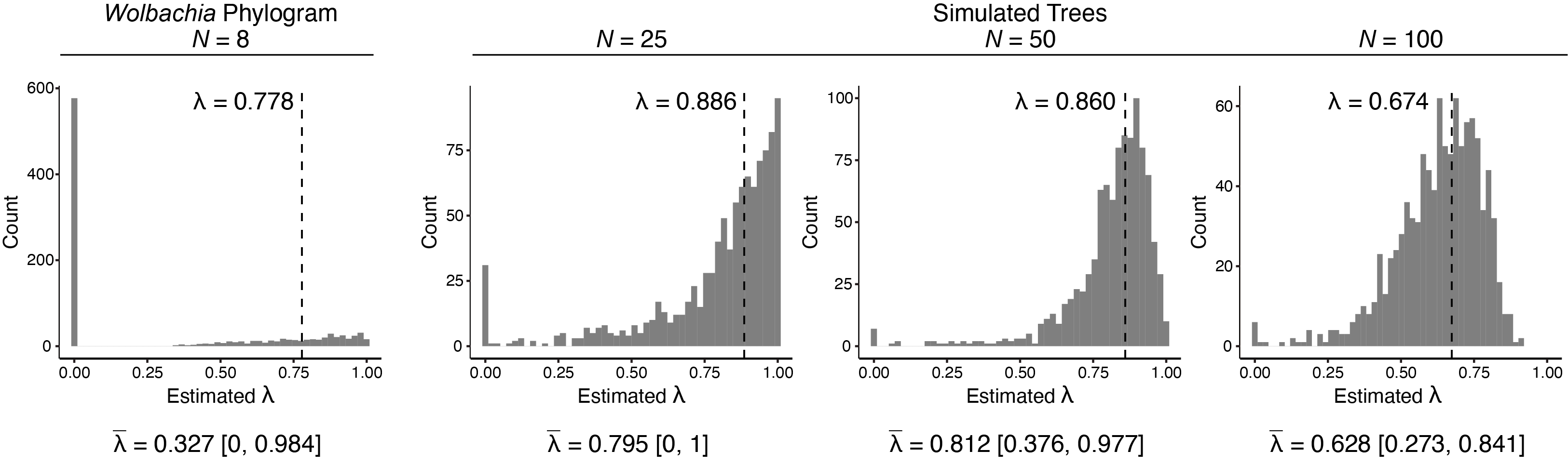


**Supplemental Figure S2.** Distribution of maximum likelihood estimates of $\lambda$ from 1,000 bootstrap replicates. The bootstrap analysis for our *Wolbachia* phylogram (Figure 2) is show to the left. To the right are simulated phylogenies with an increasing number of *Wolbachia* strains included (*N* = 25, 50, 100). For simulated trees, character evolution was simulated with our $\lambda$ estimate of 0.778 using the “sim.bdtree” and “sim.char” functions in the *geiger* R package (Harmon et al. 2008). For each graph, fitted $\lambda$ values for the original phylogeny are shown above with a vertical dashed line. Note that fitted $\lambda$ values for the simulated phylogenies differ slightly from $\lambda$ = 0.778, because “sim.char” uses a Brownian-motion model to simulate character evolution along the phylogeny. Below each graph, the mean estimate of $\lambda$ from the 1,000 replicates ($\overline{\lambda}$) is shown with associated 95% confidence intervals. The bootstrapping analyses generally show that small phylogenies (*N* = 8, 25) have a large number of near-zero $\lambda$ values arising by random chance, which increases the uncertainty of parameter estimation. Indeed, small phylogenies are likely to generate near-zero $\lambda$ values by chance, not necessarily because the phylogeny is unimportant for trait evolution (Boettiger et al. 2012). As the number of strains in our analysis increases (*N* = 50, 100), bootstrapped estimates of $\lambda$ cluster around the true $\lambda$ value fitted to the original phylogeny.
