# Supplementary material for: Pervasive Effects of Wolbachia on Host Temperature Preference
Source: mBio. 2020 Oct 6;11(5):e01768-20. doi: 10.1128/mBio.01768-20 (PMC7542361; doi:10.1128/mBio.01768-20)
Supplement: TABLE S3 [file mBio.01768-20-st003.docx]

**Supplemental Table S3.** Fly food recipe for cornmeal media. To the right, the nutritional content of the food is shown based on calculations from https://brodericklab.com/DDCC.php.

| 115 g | Inactive yeast |  | **Nutritional Values** | **g/liter of Diet** |
| --- | --- | --- | --- | --- |
| 66 g | Soy flour |  | Calories | 604.16 |
| 482 g | Cornmeal |  | Fiber | 13.15 |
| 304 g | Malt extract |  | Sugars | 34.08 |
| 38 g | Agar |  | Protein | 17.12 |
| 472 g | Dry corn syrup |  | Fat | 4.11 |
| 7.65 l | Water |  | Carbohydrates | 140.09 |
| 32.2 ml | Propionic acid |  |  |  |
| 43.4 ml | Tegosept mix |  |  |  |
