# Supplementary material for: Pervasive Effects of Wolbachia on Host Temperature Preference
Source: mBio. 2020 Oct 6;11(5):e01768-20. doi: 10.1128/mBio.01768-20 (PMC7542361; doi:10.1128/mBio.01768-20)
Supplement: TABLE S4 [file mBio.01768-20-st004.docx]

**Supplemental Table S4.** Mean temperature and standard error for each section of the custom-built thermal gradient apparatus, across all 347 experimental replicates in this study.

| **Section** | **Mean Temp. (°C)** | **Std. Error** |
| --- | --- | --- |
| 1 | 34.4 | 0.08 |
| 2 | 30.5 | 0.08 |
| 3 | 26.4 | 0.07 |
| 4 | 22.7 | 0.06 |
| 5 | 20.1 | 0.07 |
| 6 | 18.2 | 0.06 |
| 7 | 17.1 | 0.06 |
