# Supplementary material for: Pervasive Effects of Wolbachia on Host Temperature Preference
Source: mBio. 2020 Oct 6;11(5):e01768-20. doi: 10.1128/mBio.01768-20 (PMC7542361; doi:10.1128/mBio.01768-20)
Supplement: FIG S3 [file mBio.01768-20-sf003.docx]

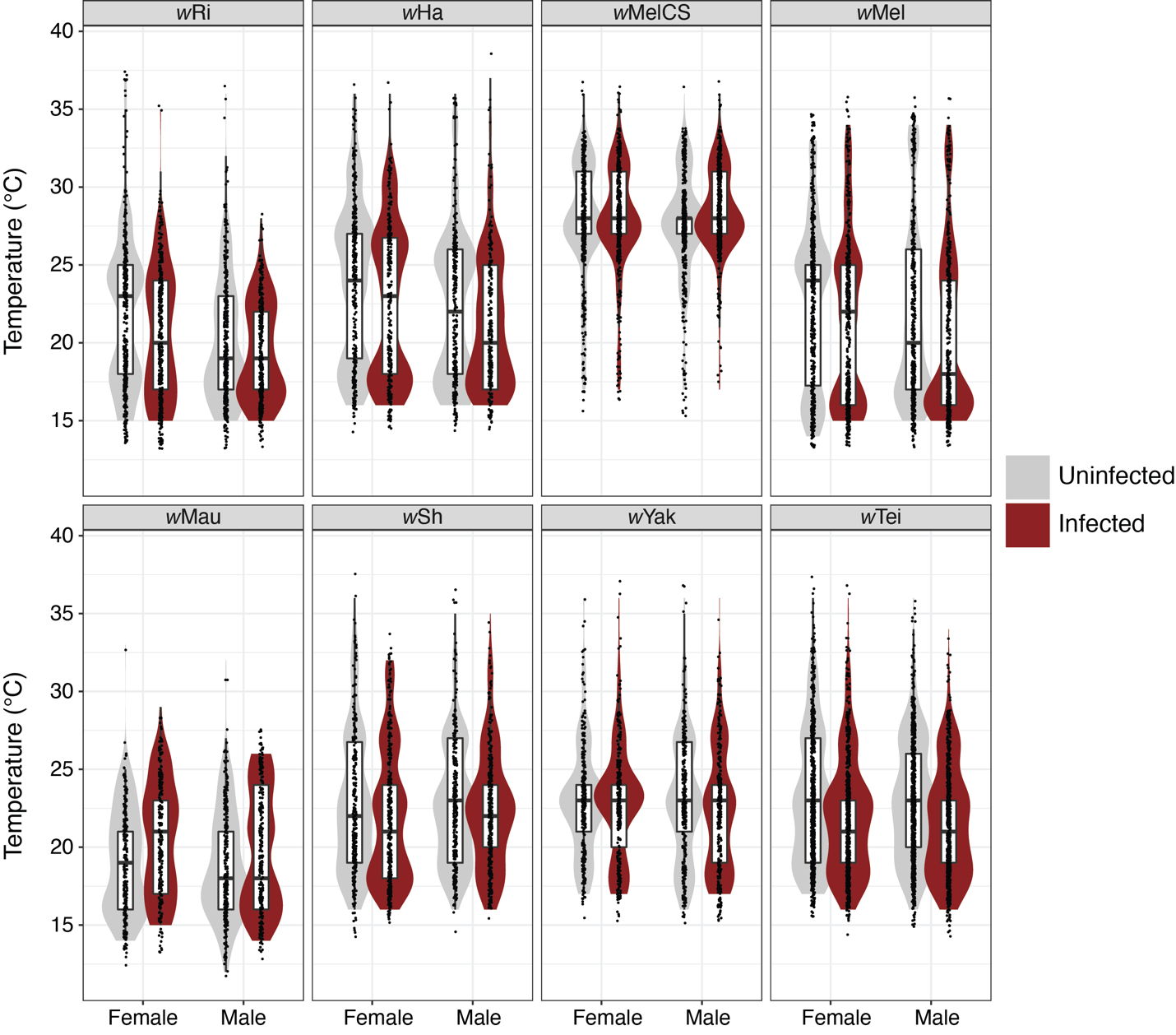


**­**

**Supplemental Figure S3.** Box plots showing temperature preference (*T_p_*) for uninfected and infected flies of each genotype when the coldest section of the thermal gradient (section 7) is included. Individual points are jittered to show overlap.
