# Supplementary material for: Pervasive Effects of Wolbachia on Host Temperature Preference
Source: mBio. 2020 Oct 6;11(5):e01768-20. doi: 10.1128/mBio.01768-20 (PMC7542361; doi:10.1128/mBio.01768-20)
Supplement: TABLE S5 [file mBio.01768-20-st005.docx]

**Supplemental Table S5.** Results and sample sizes from the GLMM and LMM analyses of *T_p_* data including the flies located in the coldest section of the thermal gradient apparatus (section 7). Statistically significant fixed effects at *P* < 0.05 are marked in bold text with asterisks.

| GLMMs |  | | |  | | |  | | |  | | |
| --- | --- | --- | --- | --- | --- | --- | --- | --- | --- | --- | --- | --- |
|  | ***w*Ri** | | | ***w*Ha** | | | ***w*MelCS** | | | ***w*Mel** | | |
| **Explanatory variable** | **coefficient** | **χ2** | ***P* value** | **coefficient** | **χ2** | ***P* value** | **coefficient** | **χ2** | ***P* value** | **coefficient** | **χ2** | ***P* value** |
| Infection Status | 0.063 | 4.757 | **0.029*** | 0.066 | 6.513 | **0.011*** | -0.022 | 1.463 | 0.226 | 0.036 | 1.788 | 0.181 |
| Sex | -0.066 | 5.115 | **0.024*** | -0.06 | 5.057 | **0.025*** | -0.007 | 0.156 | 0.693 | -0.045 | 2.743 | 0.098 |
| Age | 0.001 | 0.002 | 0.968 | 0.005 | 0.394 | 0.53 | -0.024 | 12.093 | **0.001*** | 0.008 | 0.64 | 0.424 |
| Run Order | 0.001 | 0.011 | 0.916 | 0.009 | 1.056 | 0.304 | 0.015 | 5.733 | **0.017*** | 0.02 | 5.374 | **0.02*** |
| Infection * Sex | -0.028 | 0.454 | 0.501 | -0.02 | 0.281 | 0.596 | 0.003 | 0.016 | 0.9 | 0.012 | 0.099 | 0.753 |
| Sample Size | 1534 |  |  | 1135 |  |  | 1770 |  |  | 1962 |  |  |
|  |  |  |  |  |  |  |  |  |  |  |  |  |
|  | ***w*Mau** | | | ***w*Sh** | | | ***w*Yak** | | | ***w*Tei** | | |
| **Explanatory variable** | **coefficient** | **χ2** | ***P* value** | **coefficient** | **χ2** | ***P* value** | **coefficient** | **χ2** | ***P* value** | **coefficient** | **χ2** | ***P* value** |
| Infection Status | -0.11 | 7.615 | **0.006*** | 0.047 | 5.835 | **0.016*** | 0.012 | 0.27 | 0.603 | 0.046 | 10.365 | **0.001*** |
| Sex | -0.052 | 2.055 | 0.152 | 0.018 | 0.962 | 0.327 | -0.028 | 1.45 | 0.229 | -0.017 | 1.456 | 0.228 |
| Age | -0.015 | 1.116 | 0.291 | 0.002 | 0.078 | 0.78 | 0.028 | 4.695 | **0.03*** | -0.013 | 2.965 | 0.085 |
| Run Order | 0.022 | 3.045 | 0.081 | 0.018 | 8.319 | **0.004*** | 0.01 | 1.874 | 0.171 | 0.004 | 0.849 | 0.357 |
| Infection * Sex | 0.063 | 1.383 | 0.24 | -0.026 | 0.92 | 0.337 | 0.022 | 0.450 | 0.502 | 0.016 | 0.655 | 0.418 |
| Sample Size | 1009 |  |  | 1351 |  |  | 1232 |  |  | 2951 |  |  |
|  |  |  |  |  |  |  |  |  |  |  |  |  |
| LMMs |  |  |  |  |  |  |  |  |  |  |  |  |
|  | ***w*Ri** | | | ***w*Ha** | | | ***w*MelCS** | | | ***w*Mel** | | |
| **Explanatory variable** | **coefficient** | **χ2** | ***P* value** | **coefficient** | **χ2** | ***P* value** | **coefficient** | **χ2** | ***P* value** | **coefficient** | **χ2** | ***P* value** |
| Infection Status | 1.356 | 4.142 | **0.042*** | 1.548 | 5.477 | **0.019*** | -0.604 | 1.247 | 0.264 | 0.816 | 1.736 | 0.188 |
| Sex | -1.344 | 4.077 | **0.043*** | -1.315 | 3.864 | **0.049*** | -0.189 | 0.109 | 0.741 | -0.942 | 2.307 | 0.129 |
| Age | 0.052 | 0.011 | 0.915 | 0.112 | 0.341 | 0.559 | -0.671 | 10.257 | **0.001*** | 0.174 | 0.657 | 0.418 |
| Run Order | 0.021 | 0.01 | 0.922 | 0.194 | 0.792 | 0.373 | 0.399 | 4.544 | **0.033*** | 0.435 | 4.733 | **0.03*** |
| Infection * Sex | -0.655 | 0.483 | 0.487 | -0.541 | 0.318 | 0.573 | 0.094 | 0.014 | 0.907 | 0.213 | 0.059 | 0.809 |
| Sample Size | 1534 |  |  | 1135 |  |  | 1770 |  |  | 1962 |  |  |
|  |  |  |  |  |  |  |  |  |  |  |  |  |
|  | ***w*Mau** | | | ***w*Sh** | | | ***w*Yak** | | | ***w*Tei** | | |
| **Explanatory variable** | **coefficient** | **χ2** | ***P* value** | **coefficient** | **χ2** | ***P* value** | **coefficient** | **χ2** | ***P* value** | **coefficient** | **χ2** | ***P* value** |
| Infection Status | -2.232 | 5.971 | **0.015*** | 1.03 | 4.614 | **0.032*** | 0.286 | 0.218 | 0.641 | 1.01 | 9.137 | **0.003*** |
| Sex | -1.048 | 1.611 | 0.204 | 0.392 | 0.732 | 0.392 | -0.634 | 1.112 | 0.292 | -0.376 | 1.266 | 0.261 |
| Age | -0.288 | 0.836 | 0.36 | 0.055 | 0.067 | 0.796 | 0.639 | 3.658 | 0.056 | -0.289 | 2.62 | 0.106 |
| Run Order | 0.441 | 2.326 | 0.127 | 0.397 | 6.853 | **0.009*** | 0.249 | 1.581 | 0.209 | 0.088 | 0.702 | 0.402 |
| Infection * Sex | 1.292 | 1.102 | 0.294 | -0.624 | 0.875 | 0.350 | 0.486 | 0.319 | 0.572 | 0.376 | 0.650 | 0.420 |
| Sample Size | 1009 |  |  | 1351 |  |  | 1232 |  |  | 2951 |  |  |
