# Supplementary material for: Pervasive Effects of Wolbachia on Host Temperature Preference
Source: mBio. 2020 Oct 6;11(5):e01768-20. doi: 10.1128/mBio.01768-20 (PMC7542361; doi:10.1128/mBio.01768-20)
Supplement: TABLE S6 [file mBio.01768-20-st006.docx]

**Supplemental Table S6.** The scaffold count, N50, and total assembly size of each *Wolbachia* assembly.

| **Genome** | **Host Genotype ID** | **Scaffold Count** | **N50** | **Total Assembly Size** |
| --- | --- | --- | --- | --- |
| *w*Mel | *PC75* | 76 | 29,986 | 1,237,996 |
| *w*MelCS | *Canton S Berkeley* | 89 | 25,105 | 1,233,787 |
| *w*Sh | *LD15* (Accession SRX3029362) | 83 | 25,919 | 1,294,885 |
