# Supplementary material for: Pervasive Effects of Wolbachia on Host Temperature Preference
Source: mBio. 2020 Oct 6;11(5):e01768-20. doi: 10.1128/mBio.01768-20 (PMC7542361; doi:10.1128/mBio.01768-20)
Supplement: TABLE S7 [file mBio.01768-20-st007.docx]

**Supplemental Table S7.** qPCR primers used to measure relative *Wolbachia* density in temperature shift experiments.

| **Species** | ***Wolbachia*** | **Super Group** | **Insect Locus** | **Forward Primer (5’ 🡪 3’)** | **Reverse Primer (5’ 🡪 3’)** | ***Wolbachia* Locus** | **Forward Primer (5’ 🡪 3’)** | **Reverse Primer (5’ 🡪 3’)** |
| --- | --- | --- | --- | --- | --- | --- | --- | --- |
| *D. simulans* | *w*Ri | A | *nAcRα-34E* | CTATGGTCGTTGACAGACT | GTAGTACAGCTATTGTGGC | *ftsZ* | ATCCTTAACTGCGGCTCTTG | TTCATCACAGCAGGAATGGG |
| *D. simulans* | *w*Ha | A | *nAcRα-34E* | CTATGGTCGTTGACAGACT | GTAGTACAGCTATTGTGGC | *ftsZ* | ATCCTTAACTGCGGCTCTTG | TTCATCACAGCAGGAATGGG |
| *D. melanogaster* | *w*MelCS | A | *Rpl32* | CCGCTTCAAGGGACAGTATC | CAATCTCCTTGCGCTTCTTG | *wsp* | CATTGGTGTTGGTGTTGGTG | ACCGAAATAACGAGCTCCAG |
| *D. melanogaster* | *w*Mel | A | *Rpl32* | CCGCTTCAAGGGACAGTATC | CAATCTCCTTGCGCTTCTTG | *wsp* | CATTGGTGTTGGTGTTGGTG | ACCGAAATAACGAGCTCCAG |
| *D. mauritiana* | *w*Mau | B | *nAcRα-34E* | CTATGGTCGTTGACAGACT | GTAGTACAGCTATTGTGGC | *ftsZ* | CAGAGAAGCAAGAGCGGTAG | TCTTCAAGTCCAAGCTCTGC |
| *D. sechellia* | *w*Sh | A | *Rpl32* | CCGCTTCAAGGGACAGTATC | CGATCTCCTTGCGCTTCTTG | *ftsZ* | ATCCTTAACTGCGGCTCTTG | TTCATCACAGCAGGAATGGG |
| *D. yakuba* | *w*Yak | A | *Rpl32* | CCGCTTCAAGGGACAGTATC | CGATCTCCTTGCGCTTCTTG | *ftsZ* | ATCCTTAACTGCGGCTCTTG | TTCAT CACAGCAGGAATGGG |
| *D. teissieri* | *w*Tei | A | *Rpl32* | TCGCTTCAAGGGACAGTATC | CGATCTCCTTGCGCTTCTTG | *ftsZ* | ATCCTTAACTGCGGCTCTTG | TTCATCACAGCAGGAATGGG |
